# Hypoxia pretreatment enhances the therapeutic potential of mesenchymal stem cells (BMSCs) on ozone-induced lung injury in rats

**DOI:** 10.1007/s00441-022-03627-8

**Published:** 2022-05-12

**Authors:** Shaimaa A. Abdelrahman, Abeer A. Abdelrahman, Walaa Samy, Arigue A. Dessouky, Samah M. Ahmed

**Affiliations:** 1 Medical Histology and Cell Biology Department, Faculty of Medicine, Zagazig University, Zagazig, Egypt; 2 Biochemistry and Molecular Biology Department, Faculty of Medicine, Zagazig University, Zagazig, Egypt

**Keywords:** Ozone, Lung injury, Hypoxia pretreatment, Mesenchymal stem cells, Rats

## Abstract

Ozone (O_3_) gas is a double-sided weapon. It provides a shield that protects life on earth from the harmful ultraviolet (UV) rays, but ground-level O_3_ is considered an urban air pollutant. So, a rat model of chronic O_3_ inhalation was established to assess the biochemical and morphological alterations in the lung tissue and to investigate the ameliorative effects of bone marrow–derived mesenchymal stem cells (BMSCs) with or without hypoxia pre-treatment. Forty-two adult male albino rats were divided into four groups: control, ozone-exposed, normoxic BMSC-treated, and hypoxic BMSC-treated groups. Lung tissue sections were processed for light and electron microscope examination, immunohistochemical staining for caspase 3, and iNOS. Quantitative real-time PCR for IL-1α, IL-17, TNF-α, and Nrf2 mRNA gene expression were also performed. Chronic O_3_ exposure caused elevated inflammatory cytokines and decreased antioxidant Nrf2 mRNA expression. Marked morphological alterations with increased collagen deposition and elevated apoptotic markers and iNOS were evident. BMSC treatment showed immunomodulatory (decreased inflammatory cytokine gene expression), antioxidant (increased Nrf2 expression and decreased iNOS), and anti-apoptotic (decreased caspase3 expression) effects. Consequently, ameliorated lung morphology with diminished collagen deposition was observed. Hypoxia pretreatment enhanced BMSC survival by MTT assay. It also augmented the previously mentioned effects of BMSCs on the lung tissue as proved by statistical analysis. Lung morphology was similar to that of control group. In conclusion, hypoxia pretreatment represents a valuable intervention to enhance the effects of MSCs on chronic lung injury.

## Introduction

Ozone (O_3_) is a gas made up of three oxygen atoms, as defined by The National Aeronautics and Space Administration (NASA). It is naturally found in the upper atmosphere (the stratosphere), in trace amounts to protect the earth from the dangerous ultraviolet (UV) rays of the sun (NASA 2018). The low-level ozone (tropospheric ozone) is formed near the earth’s surface by chemical reactions between sunlight and air pollutants as hydrocarbons and nitrogen oxides emitted from vehicle exhaust, gasoline vapors, and other emissions. So, O_3_ is considered as an atmospheric pollutant toxic to the living organisms in high concentrations (WHO 2003).

Ozone (O_3_) is considered as an urban air pollutant being a component of photochemical smog. Ozone pollution in urban areas is worsened either by the high populations of vehicles, which emit NO_2_ and volatile organic compounds (VOCs) or by increasing temperatures during heat waves. The ground-level ozone pollution can be 20% higher than usual in urban areas especially in the summer and autumn due to the previously mentioned reasons (Hou and Wu 2016; Sharma et al. 2016; Diem et al. 2017).

Ozone is a strong oxidizing agent; a previous study on mice showed that repeated ozone exposure caused chronic inflammation, emphysema, airflow limitation, and all features of COPD (Li et al. 2013, 2016). According to Kodavanti (2016) and Miller et al. (2016), ozone has hazardous health effects on both the lung and nervous system as it elevates cytokines and causes oxidative stress in the brain. It also activates the hypothalamic–pituitary–adrenal (HPA) axis; the main pathway in sympathetic nervous system (SNS) activation (Santiago-Lopez et al. 2010; Gackière et al. 2011).

Many experimental models of respiratory disorders such as acute respiratory distress syndrome (ARDS), idiopathic pulmonary fibrosis (IPF), cystic fibrosis (CF), bronchopulmonary dysplasia (BPD), and chronic obstructive pulmonary disease (COPD) were done searching for new lines of treatment. Mesenchymal stem cell–based therapy has demonstrated promising results in this field (Sara and Weiss 2020).

Mesenchymal stem cells (MSCs) are present in the bone marrow, adipose tissue, and umbilical cord blood (UCB). Human MSCs are multipotent, express CD73, CD90, and CD105 on their cell surfaces, and not express hematopoietic or endothelial markers as CD34, CD45, CD14, CD19, and HLA-DR. MSC-based therapeutic role is attributed to their production of trophic factors by paracrine or autocrine effects (Siqueira et al. 2010; Lv et al. 2014). Recent studies searched for new approaches to improve MSC survival, paracrine, and immunomodulatory properties by means of preconditioning of MSCs. Preconditioning means the ex vivo treatment of stem cells with chemical or physical factors to maintain and enhance their intrinsic therapeutic properties. These modifications result in better therapeutic potential with high specificity to targets than the ordinary cells (Ocansey et al. 2020).

In vivo, MSCs survive in a low oxygen tension environment (usually between 1 and 5%) (Hu et al. 2018). In vitro cultivation occurs in an average oxygen tension between 20 and 21%, which may adversely affect their cellular functions (Ocansey et al. 2020). Therefore, one of the newly used methods for maintaining and enhancing MSC functions is via hypoxia preconditioning. This method was proven to augment migratory and proliferative properties of stem cells in addition to expression of pro-survival genes and release of trophic factors by them (Kim et al. 2016; Bae et al. 2018).

Therefore, the present study aimed to produce an experimental model of lung injury in rats by chronic ozone inhalation to mimic that occurs in humans in polluted areas. Moreover, the role of hypoxia-treated BMSCs (H-BMSCs) in ameliorating the lung injury in comparison to the effect of normoxia BMSCs (N-BMSCs) is evaluated.

## Materials and methods

### Ozone (O_3_)

Ozone was created by an ozone generator at the Rheumatology Department, Faculty of Medicine, Zagazig University. A spectrometer was placed that allowed the operator to control gas flow rate and ozone concentration. The ozone flow rate was kept constant at 60 mg/ml concentration, 97% oxygen + 3% ozone gas mixture at 3 L/min.

### Isolation and cultivation of bone marrow–derived mesenchymal stem cells

Bone marrow was harvested by flushing the tibiae and femurs of 6-week-old male albino rats with Dulbecco’s modified Eagle’s medium (DMEM) supplemented with 10% fetal bovine serum. Nucleated cells were isolated with a density gradient and then re-cultured in complete culture medium supplemented with 1% penicillin–streptomycin. Cells were incubated at 37 °C in 5% humidified Co_2_ for 12–14 days until production of large colonies (80–90% confluence). The culture was washed with phosphate buffer solution (PBS) and released with 0.25% trypsin in 1 ml EDTA (5 min at 37 °C). After centrifugation, cells were re-incubated in a 50-cm^2^ culture flask (Falcon) with serum-supplemented medium (Alhadlaq and Mao 2004). MSCs in culture were characterized by their adhesiveness and fusiform shape (Rochefort et al. 2005).

### Hypoxic preconditioning of BMSCs by cobalt chloride treatment and MTT assay for cell survival

The cells were seeded in 96-well culture plates at a density of 1 × 10^4^ cells/well for 24 h. The culture medium was changed to fresh MEM containing 2% FBS and 1% antibiotics, and for hypoxic preconditioning COCl_2_ was added to the complete medium at a concentration of 100 μM; COCl_2_ was purchased from Sigma (Gabriella et al. 2018).

After 24 h, the medium was changed with a new one supplemented with 0.5 mg/ml of 3-(4,5-dimethylthiazol-2-yl)-2,5-diphenyltetrazolium bromide (MTT) for 3 h at 37 °C. The formazan produced was dissolved by solvent solution (0.1 N HCl in isopropanol), and the optical density was read at 570 nm by a microplate reader (Model 680, Bio-Rad Lab Inc., CA, USA) (Lan et al. 2015).

### Labeling of BMSCs with cell linker (PKH-26) (red fluorescence)

BM-MSCs were tagged with a fluorescence marker using Paul Karl Horan (PKH26) Red Fluorescent Cell Linker Kit (Sigma, St. Louis, Missouri, USA) prior to rat injection (Haas et al. 2000). Sections of the lung of stem cell–treated groups were examined by fluorescent microscope (Olympus BX50F4, No. 7M03285, Tokyo, Japan) at Biochemistry and Molecular Biology Department, Faculty of Medicine, Zagazig University, Zagazig, Egypt.

### Characterizations of rat BMSCs by flow cytometry

**Fig. 1:**
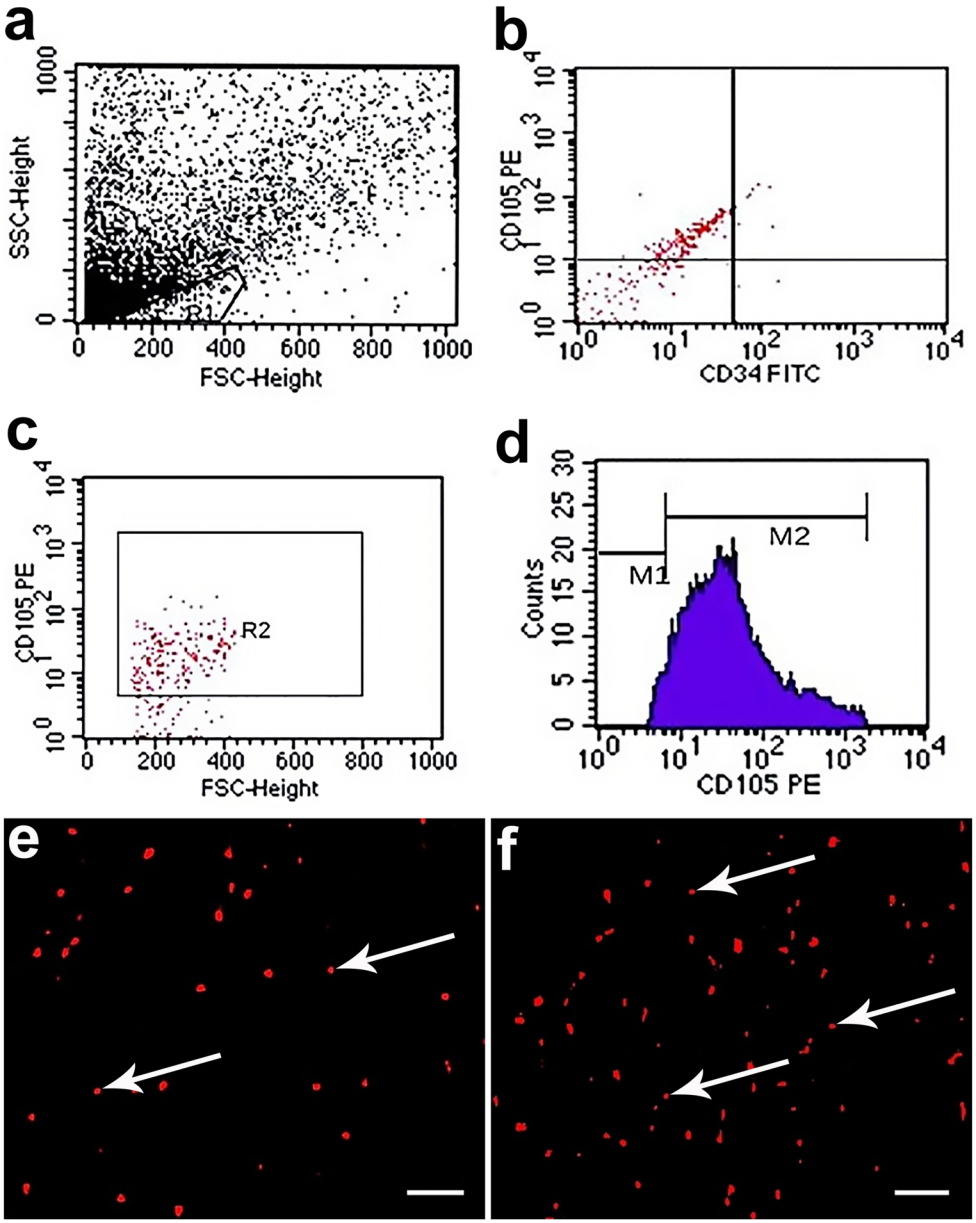
**a**–**d** Flow-cytometric analysis of the cell surface markers showing MSCs that express CD105 (mesenchymal stem cell surface marker) and do not express CD34 (hematopoietic stem cell marker). **e** PKH26-labeled stem cells appearing as bright red dots (arrow) in MSC-treated group. **f** PKH26-labeled stem cells appearing as bright red dots (arrow) in hypoxia-pretreated MSCs group indicating increased stem cells homing (fluorescent microscope × 200, scale bar 50 μm)

BMSCs were characterized by their adhesiveness and fusiform, star, or spindle shape. Flow cytometric evaluation of BMSCs was performed at Clinical Pathology Department, Faculty of Medicine, Zagazig University. BMSCs were positive for CD105 (PE labeled) cell surface expression (Barry et al. 1999) while the majority of cells showed negative expression of CD34 (FITC labeled) (Conget and Minguell 1999). Such expression pattern corresponded to BM-MSC according to the International Society of Cellular Therapy System (Fig. 1a).

### Experimental animals

Forty-two healthy adult male Wistar albino rats (weighing 180–200 g) were obtained and maintained at the Breeding Animal House of the Faculty of Medicine, Zagazig University, Egypt. Animals were kept for acclimatization in plastic cages with a stainless-steel wire-bar lid at a controlled temperature (23 ± 1 °C) and humidity (55 ± 5%) in an artificially illuminated room (12-h light/12-h dark cycle), completely free from chemical contamination. They were fed with standard laboratory food and allowed to access it and drink water freely. All experimental procedures were performed in accordance with the guidelines of the Institutional Animal Care and Use Committee and accepted by Faculty of Medicine, Zagazig University.

### Experimental design

The animals were divided into four groups as follows:

#### **Group I (control group)**:

This group included 12 rats, divided into two equal subgroups:

- Subgroup Ia (*n* = 6): Rats received no treatment and served as negative control.
- Subgroup Ib (*n* = 6): received 300 μl cell-free phosphate buffered saline (PBS) by IV injection in the tail vein then they were left until the end of the experiment.

#### Group II (ozone-exposed rats):

Included 10 rats that were exposed to 1 ppm ozone by inhalation, 5 h/day, 3 consecutive days/week for 13 weeks (Miller et al. 2016).

#### Group III (N-BMSC-treated rats):

Included 10 rats that were exposed to 1 ppm ozone by the same route and for the same duration as in group II, then each rat was injected with N-BMSCs at a dose of 1 × 10^6^ cells in 300 μl PBS by IV injection in the tail vein (Wei et al. 2012).

#### Group IV (H-BMSC-treated rats):

Included 10 rats that were exposed to 1 ppm ozone by the same route and for the same duration as in group II, then each rat was injected with H-BMSCs at a dose of 1 × 10^6^ cells in 300 μl PBS by IV injection in the tail vein (Wei et al. 2012).

At the end of experiment, 4 weeks after MSC or PBS injection, animals from all groups were fasted overnight then they were anaesthetized with an intraperitoneal injection of 75 mg/kg ketamine (El Bana and Shawky 2019). The chest was opened and specimens of the left lung of each rat of all groups were divided into two parts. Sections of the first part were processed for light and electron microscope examinations. Sections of the second part were stored at − 80 °C for biochemical study.

### Gene expression analysis by RT-qPCR for IL-1α, IL-17, TNF-α, and Nrf2 mRNA gene expression

Total RNA was prepared from the lung tissues using RNeasy Mini Kit (Qiagen). Complementary DNA (cDNA) was synthesized using a cDNA reverse transcription kit (TIAGEN FastQuant RT Kit) following the manufacturer’s instruction. Quantitative PCR analysis was performed using SYBR Green PCR Master Mix Reagent (QuantiTect SYBR Green PCR Kits; Qiagen). The thermal cycling program of qPCR was as follows: step 1, 15 min at 95 °C; step 2, 15 s at 95 °C; step 3: 30 s at 60 °C; step 4: 30 s at 72 °C, with step 2 to step 4 repeated for 40 cycles.

Relative gene expression of mRNA of IL-1α, IL-17, TNF-α, and Nrf2 was normalized to β-actin expression. Relative changes in gene expression were calculated using the 2 − ΔΔCT method. Sequences of primers used in the PCR (synthesized by Invitrogen, Thermo Fisher Scientific) are listed in Table 1.

**Table 1:** Primer sequence for IL-1α, IL-17, TNF-α, and Nrf2

Primer	Forward primer	Reverse primer
IL-1α	5′-TTGAAGACCTAAAGAACTGTTACAGTGAA-3′	5′-GCCATAGCTTGCATCATAGAAGG-3′
IL-17	5′-CCTGGCGGCTACAGTGAAG-3′	5′-TTTGGACACGCTGAGCTTTG-3′
TNF-α	5′-AGCCGATGGGTTGTACCTTGTCTA-3′	5′-TGAGATAGCAAATCGGCTGACGGT-3′
Nrf2	5′-TGAAGCTC AGCTCGCATTGA-3′	5′-TGCTCCAG CTCGACAATGTT-3′
β-Actin	5′-TGACCGAGCGTGGCTACAG-3′	5′-GGGCAACATAGCACAGCTTCT-3′

### Histological study

This work was carried out in Medical Histology and Cell Biology Department, Faculty of Medicine, Zagazig University, Egypt.

#### Light microscope study

Specimens for light microscopy were fixed in 10% buffered formol saline for 24 h and processed to prepare 5-μm-thick paraffin sections for staining with hematoxylin & eosin (H&E) and Mallory trichrome stains (Bancroft and Layton 2013).

#### Immunohistochemical study

Immunohistochemical expression of CD105, anti-caspase-3 antibody, and iNOS was carried out using streptavidin–biotin complex immunoperoxidase system (Ramos-Vara et al. 2008). Serial sections of paraffin-embedded specimens were deparaffinized on charged slides. The sections were incubated in 0.1% hydrogen peroxide for 30 min to block the endogenous peroxidase and then incubated with the primary antibody.

For CD105 detection, sections were incubated with rat anti-mouse CD105 monoclonal antibody, eBioscience (Cat. No. 14–1051-82, 1:200 dilution; Thermo Fisher Scientific, Rockford, USA).

For anti-caspase-3 immunostaining, sections were incubated with rabbit polyclonal IgG anti-caspase-3 antibody (Cat. No. PA5-16,335; Thermo Fisher Scientific, Rockford, USA) diluted at 1:200 in PBS for 30 min at room temperature.

For iNOS immunostaining, sections were incubated overnight at 4 °C with rabbit polyclonal antibody to iNOS (Cat. No. PA3-030A; Thermo Fisher Scientific, Rockford, USA) diluted at 1:100.

After several washes with PBS, primary antibodies were detected by incubation with biotinylated goat anti-mouse and anti-rabbit antibodies (Zymed Laboratories; South San Francisco, CA) for 30 min at room temperature. Thereafter, all sections were incubated with the streptavidin–biotin peroxidase complex for 30 min at room temperature. After washing with PBS, reactions were visualized with 30, 30-diaminobenzidine-tetrahydrochloride (DAB—Sigma-Aldrich Chemical Co., St. Louis) used as a chromogen to visualize antibody binding. The sections were counterstained with Mayer’s hematoxylin, dehydrated, and mounted. For negative control, the primary antibody was replaced with PBS. Sections were examined and photographed with a microscope (Leica, Germany).

#### Electron microscope study

Specimens for electron microscopy were immediately fixed in 2.5% phosphate buffered glutaraldehyde (pH 7, 4), post-fixed in 1% osmium tetroxide in the same buffer at 4 °C, dehydrated, and embedded in epoxy resin. Ultrathin sections were obtained (Leica ultracut UCT), stained with uranyl acetate and lead citrate, examined, and photographed (JEOL JEM-2100) using a transmission electron microscope (Jeol Ltd., Tokyo, Japan) in the Electron Microscope Research Unit, Faculty of Agriculture, Mansoura University, Egypt (Glauret and Lewis 1998; Hayat 2000).

#### Histomorphometric study

The image analyzer computer system Leica Qwin 500 (Leica Imaging System, Ltd., Cambridge, England) was used to evaluate the percentage of each parameter. The data were analyzed by Leica Qwin 500 software with the aid of a digital camera connected to an optical microscope (Olympus, Tokyo, Japan) in the Pathology Department, Faculty of Dentistry, Cairo University, Cairo, Egypt. Ten non-overlapping fields were randomly chosen from each rat in each group and the means of the measurements of the parameter in each section were recorded for each animal. Examined measures included.

- Area percentage (%) of collagen fibers in Mallory trichrome-stained sections.
- Area percentage (%) of positive immunoreaction for caspase 3 and iNOS immunoperoxidase stained sections.

### Statistical analysis

Data were expressed as means ± standard deviation. Analysis was done using Statistical Package for Social Sciences (SPSS) version 22.0 (IBM Corp., Armonk, NY, USA). One-way analysis of variance (ANOVA) was used, followed by Tukey’s honestly significant difference (Tukey’s HSD) test as a post hoc test. The probability values (*P*) less than 0.05 were considered significant and highly significant when the *P* values were less than 0.001.

## Results

### Flow cytometry results

By flow cytometry, BMSCs expressed mesenchymal stem cell surface marker CD105 and did not express hematopoietic stem cell marker CD34 as shown in Fig. 1a–d.

### Detection of MSCs homing in lung tissue

**Fig. 2:**
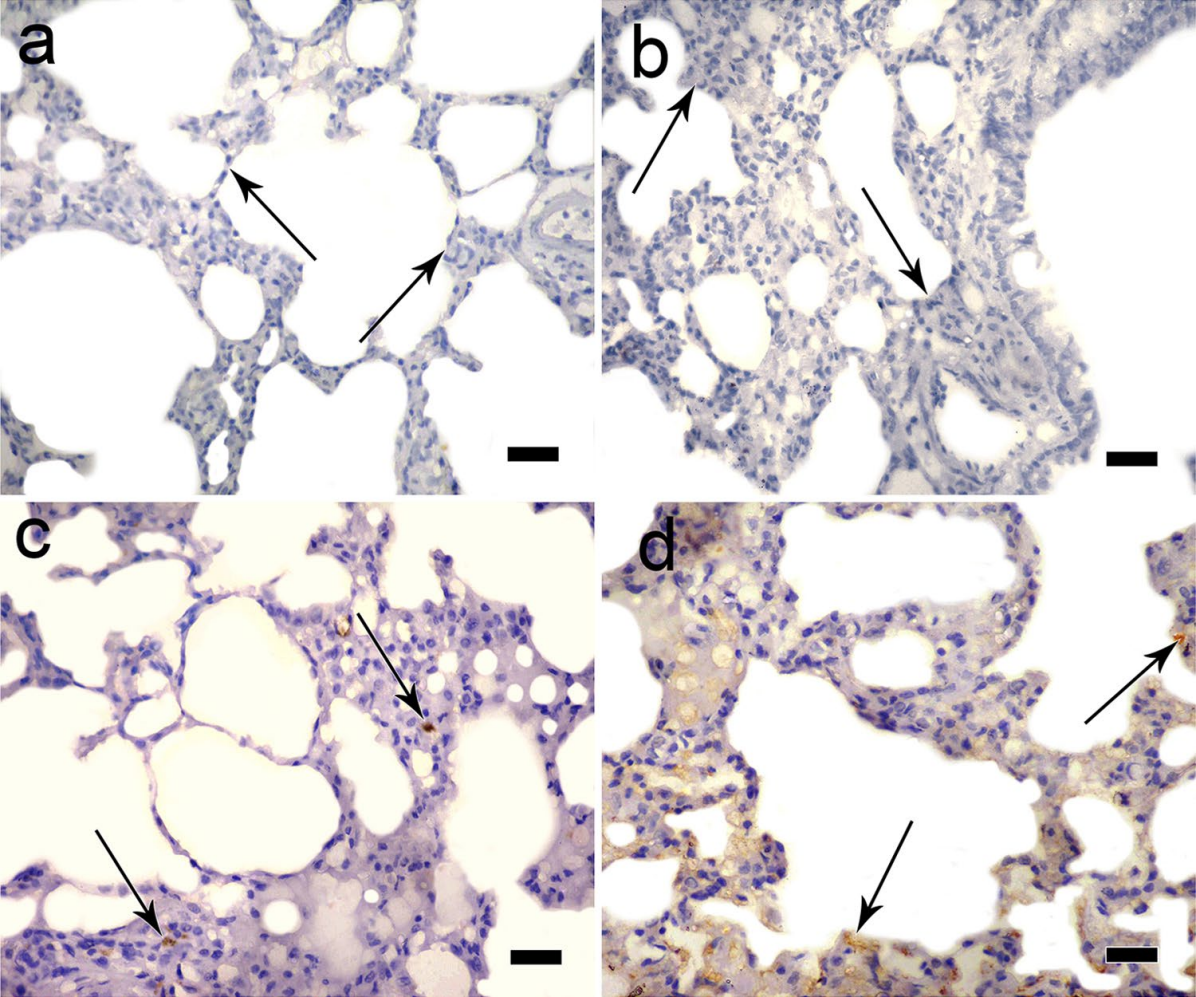
Immunohistochemical staining for CD105-positive stem cells showing negative results in control and ozone-treated groups (**a** and **b**, respectively), and positive immunostaining for CD105 in MSC-treated groups with more homing in hypoxia-pretreated MSC group **(d)** than normoxic MSC group **(c)**

- Sections in the rat lung of MSC-treated groups examined by fluorescent microscope showed PKH26-labeled stem cells that appeared as bright red dots with more homing in hypoxia-pretreated MSC group (Fig. 1f) than normoxic MSC group (Fig. 1e).
- Immunohistochemical staining for CD105-positive stem cells showed negative results in control and ozone-treated groups (Fig. 2a and b, respectively), and positive immunostaining for CD105 in MSC-treated groups with more homing in hypoxia-pretreated MSC group (Fig. 2d) than normoxic MSC group (Fig. 2c).

### Effect of hypoxia pre-treatment on MSC survival

**Fig. 3:**
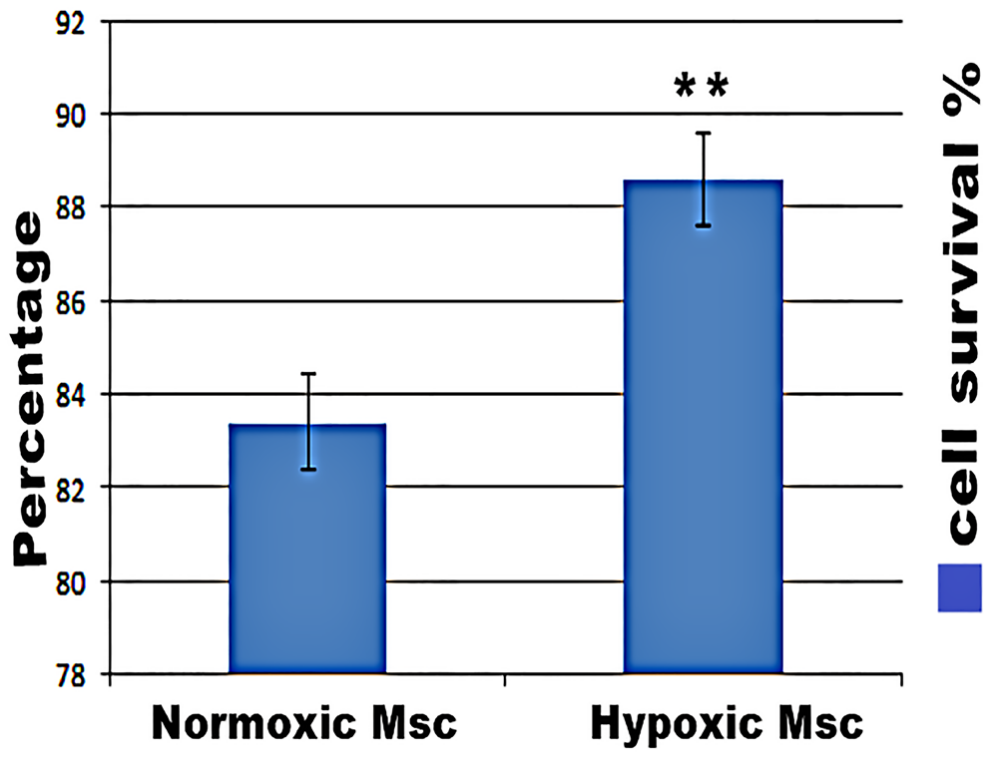
MTT assay for detection of cell survival in MSC-treated groups showing a highly significant (*P* value < 0.0001) increase in cell survival of MSC samples pre-treated with CoCl_2_ for induction of hypoxia in culture medium than normoxic MSC culture only

The effect of hypoxic preconditioning on the survival period of the MSCs was determined by MTT assay (Fig. 3). We found a highly significant (*P* value < 0.0001) increase in cell survival of MSC samples pre-treated with CoCl_2_ for induction of hypoxia in culture medium (88.6 ± 1.78) than normoxic MSC culture only (83.4 ± 1.62).

### Results of gene expression

**Fig. 4:**
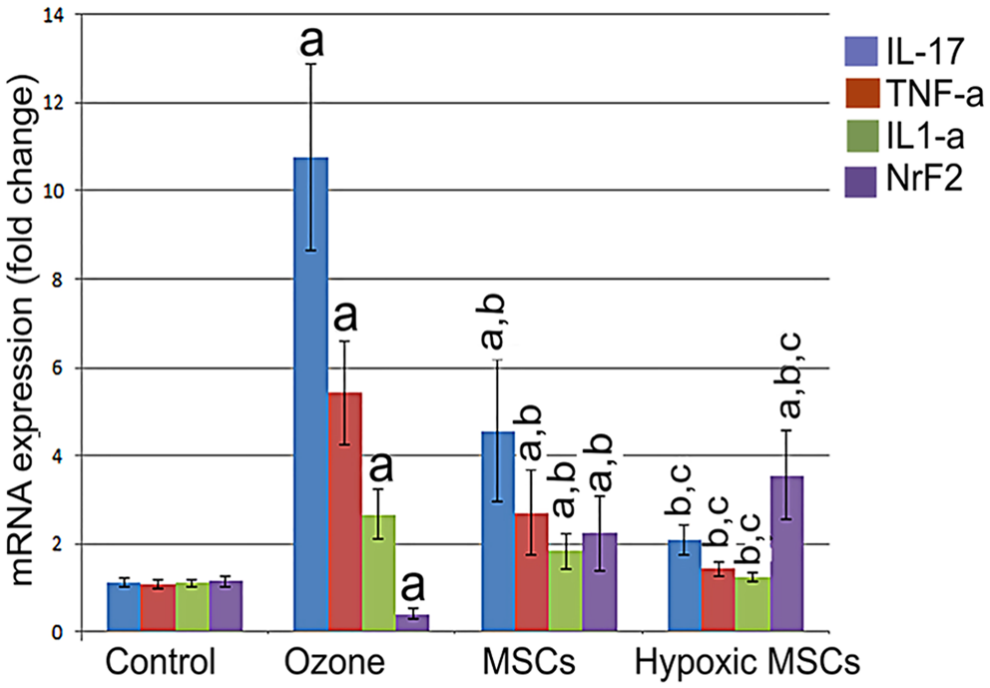
Results of gene expression of IL-17, IL-1α, TNF-α, and Nrf2. **a** Significant when compared to control group. **b** Significant when compared to ozone group. **c** Significant when compared to MSC group

In the current study, we reported that ozone exposure is associated with highly significant increase (*P* < 0.001) in the gene expression of the inflammatory cytokines IL1-α, IL17, and TNF-α while there was a highly significant decrease (*P* < 0.001) in Nrf2 gene expression as a result of oxidative stress and imbalance of antioxidant system. However, in the MSC-treated groups (either hypoxic or normoxic), there was a highly significant decrease (*P* < 0.001) in the expression of IL1, IL17, and TNF-α in addition to highly significant Nrf2 (*P* < 0.001) upregulation. Moreover, in hypoxic MSCs, there was no significant difference in the expression of IL1-α, IL17, and TNF-α when compared to the control group indicating the beneficial effects of hypoxic MSCs over the normoxic MSCs (Fig. 4).

### Histological results

**Fig. 5:**
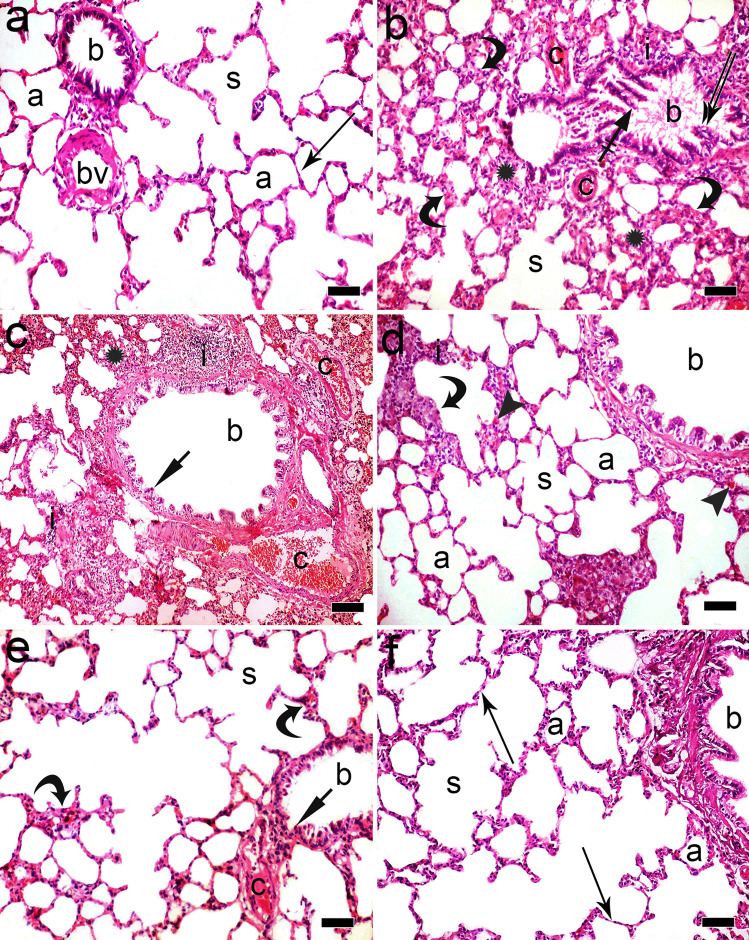
A photomicrograph of H&E-stained sections in rat lung **a** control group shows the normal lung histological structure; bronchioles (b), polygonal alveoli (a) that were separated by thin interalveolar septae (arrow), alveolar sacs (s), and the interstitium with blood vessels (bv). **b** Ozone-treated group reveals marked affection as bronchioles (b) are lined by epithelial cells with dark stained nuclei and vacuolated cytoplasm (double arrow). Desquamated epithelial lining (crossed arrow) in the bronchiolar lumen, collapsed alveoli (asterisk) with thickened interalveolar septae (curved arrow), congested blood vessels (c), and interstitial inflammatory cellular infiltration (i) are also seen. Notice alveolar sac (s). **c** Some sections of ozone-treated group showing markedly congested blood vessels (c) and inflammatory cellular infiltration (i) in the interstitium. A bronchiole (b) has epithelial cells with dark stained nuclei (short arrow). Collapsed alveoli (asterisk) are also seen. **d** MSC-treated group showing improvement in the histological structure of the lung tissue; normal bronchioles (b), alveoli (a), and alveolar sacs (s). Some alveoli have thick interalveolar septae (curved arrow), extravasated blood (arrowhead) in the interstitium, and inflammatory cellular infiltration (I) are also seen. **e** Other sections in MSC-treated group still have bronchioles (b) that are lined by epithelium with dark stained nuclei (short arrow), alveolar sacs (s), alveoli (a) with thick interalveolar septae (curved arrow), and congested blood vessels (c) in the interstitium. **f** Hypoxic MSC group showing apparently normal bronchioles (b), alveolar sacs (s), and alveoli (a) that are separated by thin interalveolar septae (arrow) (H&E × 200, scale bar 50 μm)

Examination of H&E-stained sections of the control lung showed normal lung histological structure; bronchioles, polygonal alveoli that were separated by thin interalveolar septae, alveolar sacs, and the interstitium contained blood vessels (Fig. 5a). Ozone-treated group revealed marked affection of lung tissue as the bronchiolar epithelium showed dark-stained nuclei and vacuolated cytoplasm in addition to desquamated epithelial cells in the bronchiolar lumen. The alveoli were collapsed with thickened interalveolar septae. The interstitial tissue showed congested blood vessels, inflammatory cellular infiltration, and extravasated blood in some sections (Fig. 5b, c). Normoxic MSC-treated group showed improvement in the histological structure of the lung tissue as some sections showed normal bronchioles, alveoli, and alveolar sacs; however, other sections still revealed dark-stained nuclei in bronchiolar epithelium, thickened interalveolar septae, congested blood vessels, and inflammatory cellular infiltration (Fig. 5d, e). In hypoxic MSC-treated group, apparently normal alveoli and alveolar sacs were separated by thin interalveolar septae in addition to normal shaped bronchioles (Fig. 5f).

**Fig. 6:**
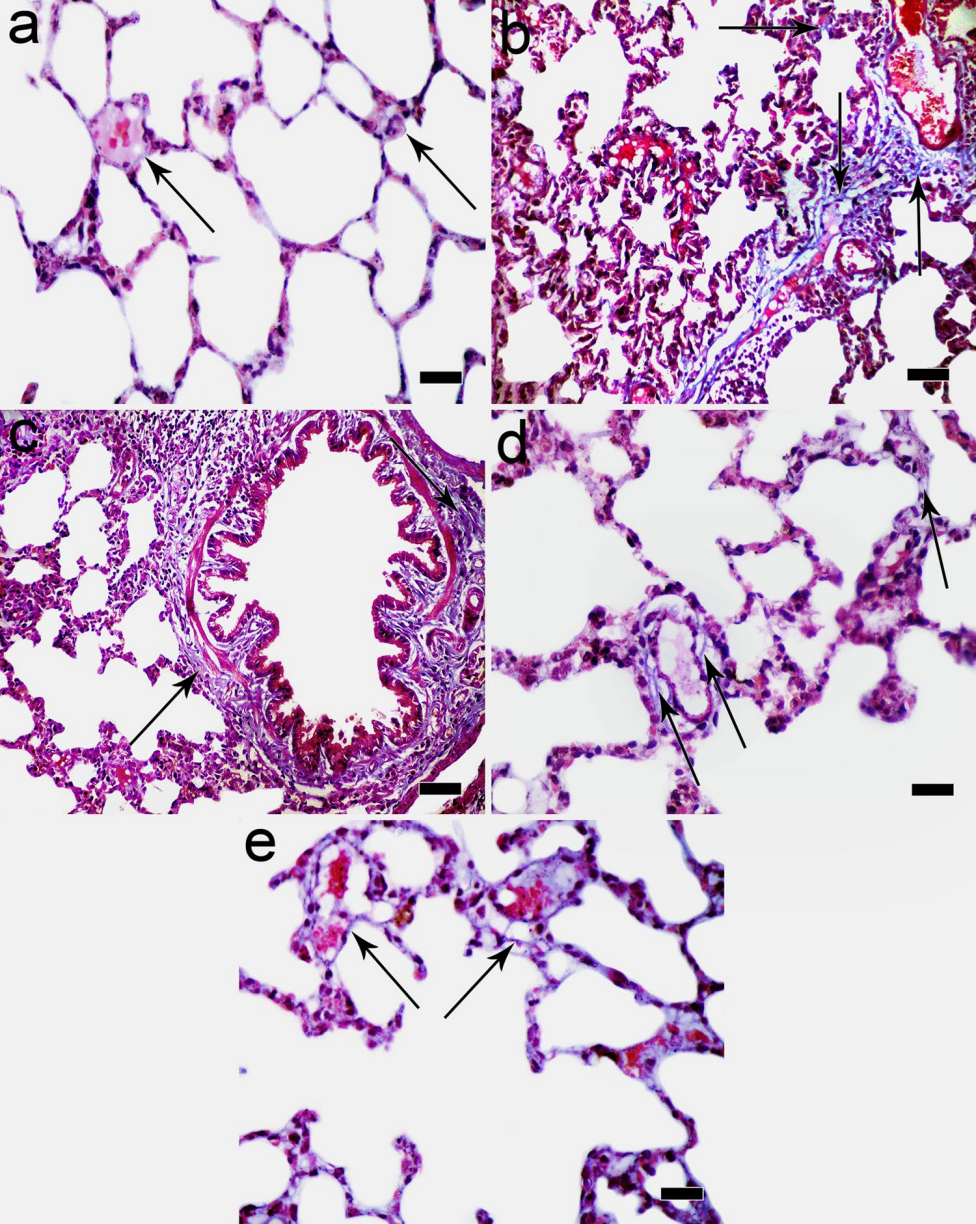
**a** Photomicrograph of Mallory trichrome–stained sections in the control lung showing minimal collagen fibers (arrow) in the interstitium around blood vessels. **b** Increased collagen fibers (arrow) in the interstitium between alveoli and around blood vessels in ozone-treated group. **c** Excessive collagen fibers in the interstitium and around bronchioles (arrow) are also noticed in ozone-treated group. **d** Some collagen fibers (arrow) are detected surrounding blood capillaries and in the interstitium between alveoli in MSC-treated group. **e** Few collagen fibers (arrow) are detected in hypoxic MSC group (Mallory trichrome × 400, scale bar 20 μm)

Mallory trichrome-stained sections of the control lung showed minimal collagen fibers in the interstitium around blood vessels (Fig. 6a), while excessive collagen fibers were detected in the interstitium between alveoli, around blood vessels, and around bronchioles in ozone-treated group (Fig. 6b, c). In normoxic MSC-treated group, some collagen fibers were detected surrounding blood capillaries and in the interstitium between alveoli (Fig. 6d), but they were markedly decreased in hypoxic MSC group (Fig. 6e).

### Immunohistochemical results

**Fig. 7:**
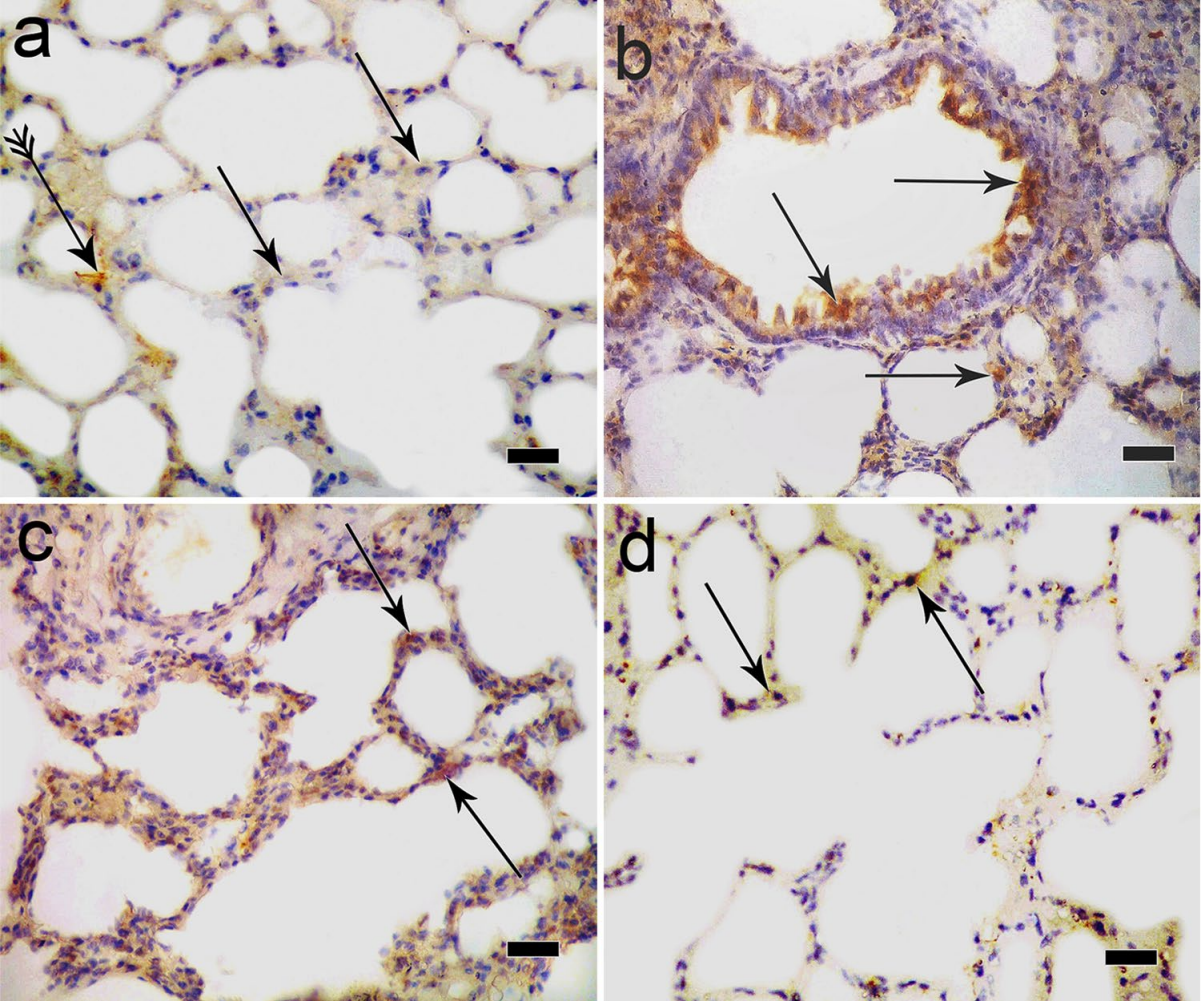
**a** Photomicrograph of immunoperoxidase technique for caspase 3 immunoreaction of the control group showing negative reaction in many alveolar cells cytoplasm (arrow) while faint positive immunoreaction appeared in the cytoplasm of few alveolar cells (crossed arrow). **b** Sections of ozone-treated group reveal a strong positive immunoreaction in the cytoplasm of lung epithelial cells and bronchiolar epithelium (arrow). **c** In MSC-treated group, there was a positive immunoreaction in the cytoplasm of some alveolar epithelial lining (arrow).* d* However, in hypoxic MSC group, weak positive immunoreaction in the cytoplasm of alveolar epithelial lining is also noticed (arrow) (immunoperoxidase technique × 400, scale bar 20 μm)

Immunoperoxidase technique for caspase 3 immunoreaction of the control group showed faint positive reaction in the cytoplasm of alveolar epithelial lining (Fig. 7a). Ozone-treated group revealed a strong positive reaction in the cytoplasm of lung epithelial cells and bronchiolar epithelium (Fig. 7b). Normoxic MSC-treated group showed positive reaction in the cytoplasm of some alveolar epithelial lining (Fig. 7c); however, in hypoxic MSC group, a weak reaction was noticed in the cytoplasm of alveolar epithelial lining (Fig. 7d).

**Fig. 8:**
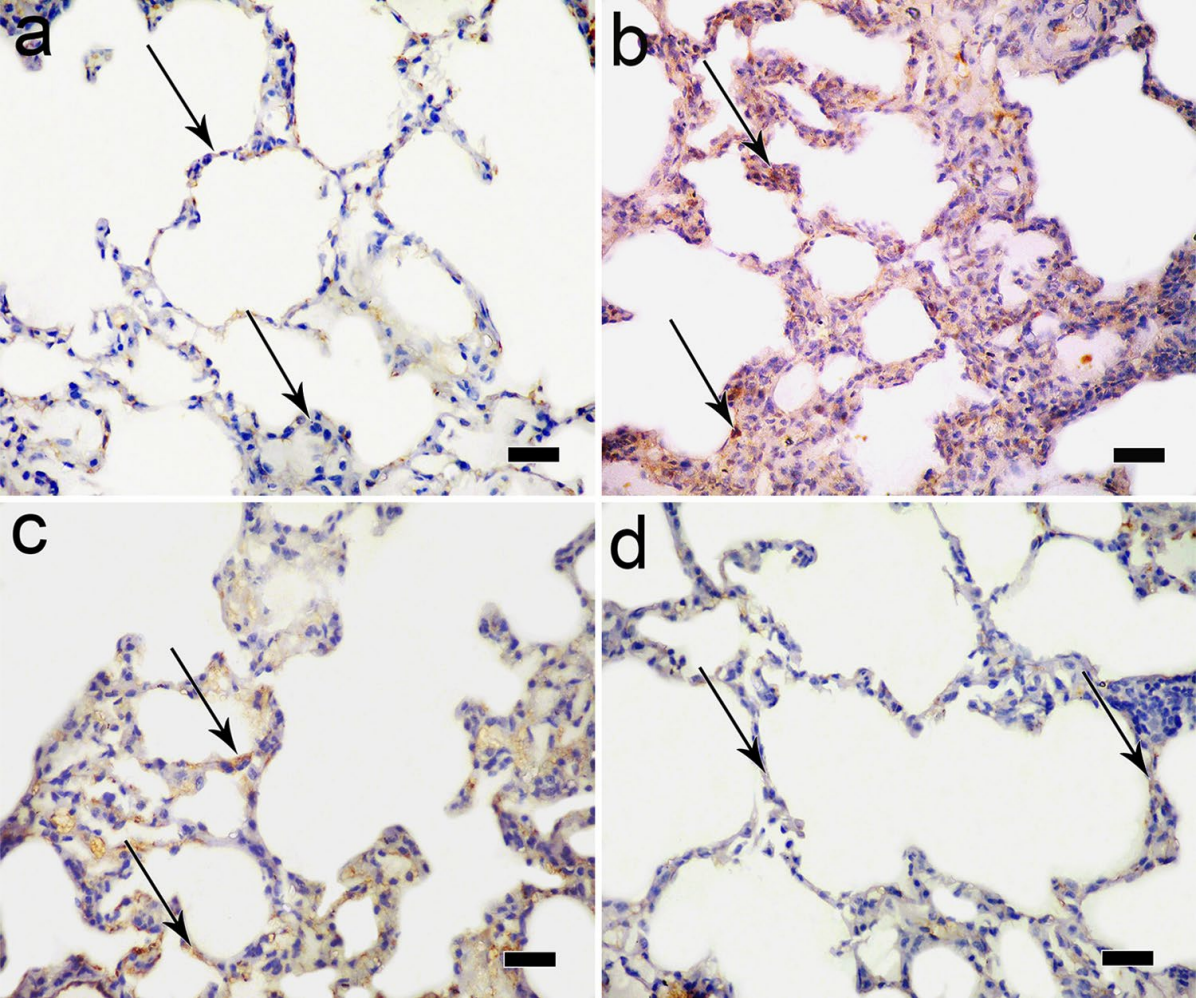
**a** Photomicrograph of immunoperoxidase technique for iNOS of the control group showing negative immunoreaction in the cytoplasm of alveolar epithelial lining (arrow). **b** Ozone-treated group reveals a strong positive immunoreaction in the cytoplasm of lung epithelial cells (arrow). **c** In MSC-treated group, weak positive immunoreaction in the cytoplasm of some alveolar epithelial lining (arrow). **d** However, hypoxic MSC group showing negative immunoreaction in the cytoplasm of alveolar epithelial lining cells is detected (arrow) (immunoperoxidase technique × 400, scale bar 20 μm)

Immunoperoxidase technique for iNOS of the control group revealed negative immunoreaction in the cytoplasm of alveolar epithelial cells (Fig. 8a). Ozone-treated group revealed a strong positive reaction in the cytoplasm of lung epithelial cells (Fig. 8b). Normoxic MSC-treated group showed a weak positive reaction in the cytoplasm of some alveolar epithelial lining (Fig. 8c); however, hypoxic MSC group showed a negative immunoreaction in the cytoplasm of alveolar epithelial lining cells (Fig. 8d).

### Ultrastructural results

**Fig. 9:**
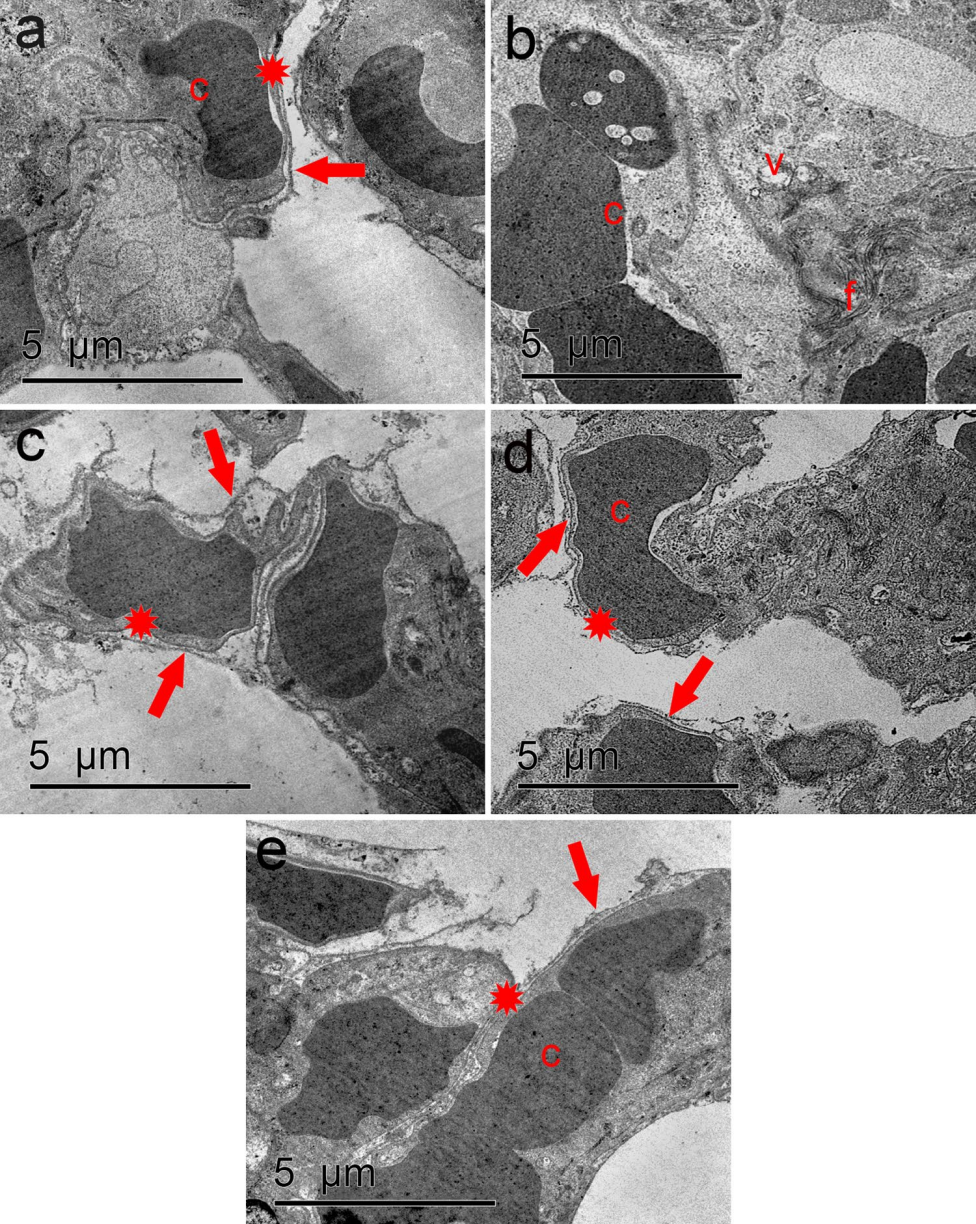
**a** Electron micrograph of the control group showing the blood air barrier (asterisk) that was formed of type I pneumocyte processes (arrow), fused basal laminae of pneumocyte type I and blood endothelium and endothelial cell cytoplasm (c). **b** Ozone-treated group reveals congested capillaries (c), collagen fibers (f), and vacuolations in the interstitial cells (v). **c** Other sections in the same group showing thick (asterisk) and corrugated (arrow) blood air barrier. **d** MSC-treated group showing disrupted blood air barrier (asterisk) with discontinuous type I pneumocyte processes (arrow). **e** In hypoxic MSC group, the blood air barrier appeared normal (asterisk) with intact type I pneumocyte processes (arrow). Blood capillaries (c) can be seen **(**TEM; scale bar, 5 μm)

Ultrathin sections of the control lung showed the blood air barrier that was formed of type I pneumocyte processes, fused basal laminae of pneumocyte type I, and blood endothelium and endothelial cell cytoplasm (Fig. 9a). Ozone-treated group revealed severely affected lung tissue in the form of thick and corrugated blood air barrier, congested capillaries, excess collagen fibers, and vacuolations in the interstitial cells (Fig. 9b, c). Normoxic MSC-treated group revealed also disrupted blood air barrier (formed of type I pneumocyte processes, discontinuous basal laminae of pneumocyte type I and blood endothelium, and endothelial cell cytoplasm) (Fig. 9d). In hypoxic MSC group, the blood air barrier appeared normal shaped similar to control (Fig. 9e).

**Fig. 10:**
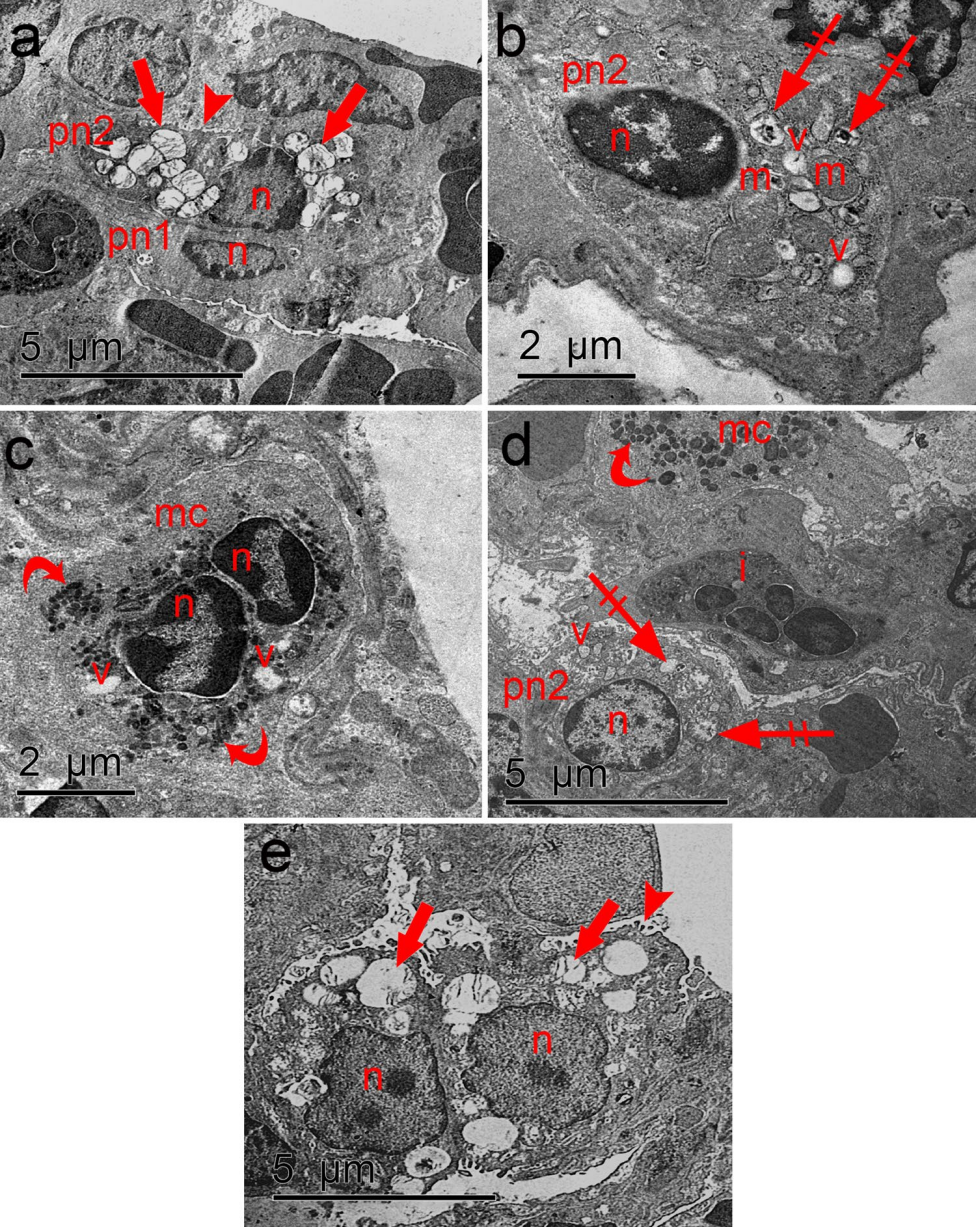
**a** Electron micrograph of the control group showing pneumocyte type II (pn2) of the control group having surface microvilli (arrowhead), euchromatic nucleus (n) with some peripheral heterochromatin, and numerous lamellar bodies (arrow). Pneumocyte type I (pn1) is noticed with elongated nucleus (n). **b** Pneumocyte type II (pn2) of ozone-treated group has heterochromatic nucleus (n), mitochondria with irregular cristae (m), vacuoles (v), and lamellar bodies with electron-dense material (crossed arrow). **c** Some sections in ozone-treated group reveal alveolar macrophage (mc) having heterochromatic nuclei (n), electron-dense bodies (curved arrow), and some vacuoles (v). **d** MSC-treated group reveals pneumocyte type II (pn2) with euchromatic nucleus (n) and numerous lamellar bodies containing electron-dense material (crossed arrow) and some vacuolations (v). Alveolar macrophage (mc) has electron-dense bodies (curved arrow). Interstitial cell (i) is also seen. **e** In hypoxic MSC group, pneumocyte type II (pn2) has surface microvilli (arrowhead), euchromatic nuclei (n), and numerous lamellar bodies (arrow) (TEM; scale bar **a**, **d**, **e**: 5 μm; **b**, **c**: 2 μm)

Pneumocyte type II of the control group showed surface microvilli, euchromatic nucleus with some peripheral heterochromatin, and numerous lamellar bodies (Fig. 10a). Pneumocyte type I was noticed with elongated nucleus. Pneumocyte type II of ozone-treated group showed cellular injury in the form of heterochromatic nucleus, mitochondria with irregular cristae, vacuoles, and lamellar bodies with electron-dense material. Alveolar macrophage was also detected in electron micrographs of the same group with characteristic heterochromatic nuclei and electron-dense bodies and showed some vacuoles (Fig. 10b, c). MSC-treated groups revealed normal appearance of pneumocyte type II with surface microvilli, euchromatic nucleus, and numerous lamellar bodies containing electron-dense material. Alveolar macrophage with electron-dense bodies and interstitial cells were also detected (Fig. 10d and e, respectively).

### Histomorphometry results

**Fig. 11:**
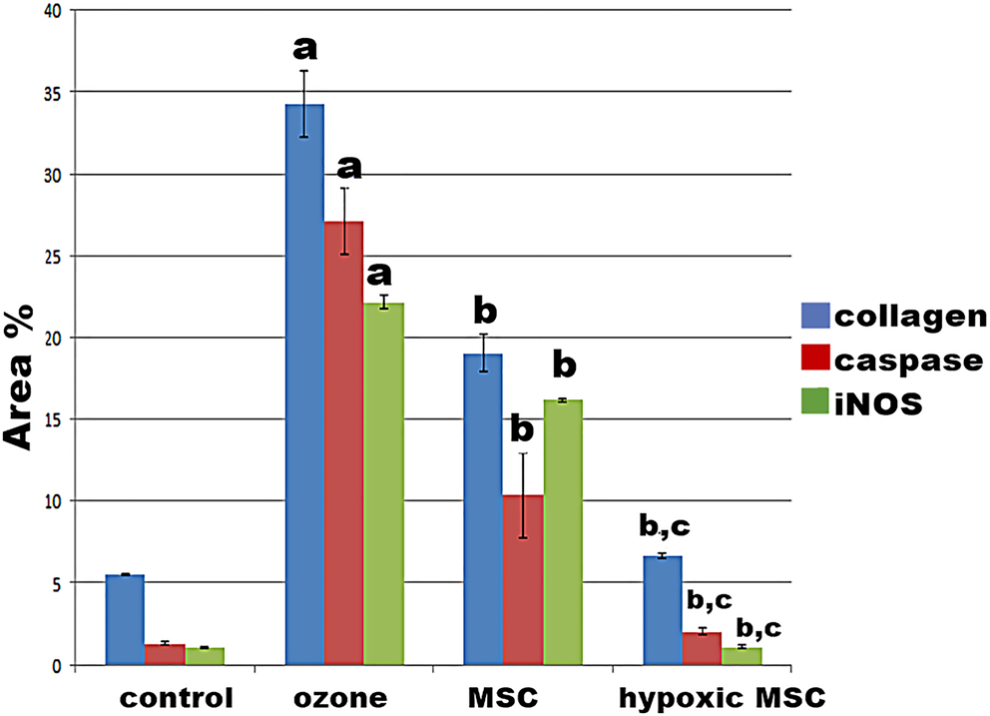
Area % of collagen fibers, caspase 3, and iNOS in the different studied groups. **a** Significant when compared to control group. **b** Significant when compared to ozone group. **c** Significant when compared to MSC group

- High statistically significant increases (*P* < 0.001) in the mean area % of collagen fibers and caspase 3 immunoreaction were detected in ozone-treated group as compared to the control and hypoxic MSCs groups. A statistically significant increase (*P* < 0.01) in the mean area percent of iNOS immunoreaction was noticed in ozone-treated group as compared to the control and hypoxic MSC groups.
- Statistically significant differences were detected between the two MSC groups concerning all measured parameters while no statistically significant differences were detected between control and hypoxic MSC groups (Fig. 11).

## Discussion

Ozone is a major air pollutant, with progressively rising levels due to ongoing global warming. As the respiratory epithelium is the first structure exposed to the inhaled O_3_, thus continuous studies are required to elucidate the mechanisms of O_3_-induced lung injuries and to find effective treatments for such injuries. So, the aim of the present study was to establish an experimental model of lung injury induced by chronic ozone exposure and to compare the therapeutic efficacy of normoxic versus hypoxia preconditioned MSCs in ameliorating the resultant injury.

In the current study, chronic O_3_ exposure led to a significant upregulation of inflammatory cytokine (IL-1α and -17 and TNF-α) gene expression, with a significant downregulation of antioxidant Nrf2 gene expression. Our results were in accordance with that of Nawijn et al. (2011). They mentioned that epithelial cell damage by O_3_ causes protein leakage into BALF with the release of inflammatory mediators as IL-1α, IL-1β, IL-25, IL-33, TSLP, leukotrienes, prostaglandins, and chemokines that attract neutrophils, monocytes, and lymphocytes. They added that lamina propria cells, vascular endothelium, fibroblasts, and smooth muscle cells also are targets of O_3_-induced oxidative stress. The pro-inflammatory cytokines IL-17A and IL-1β were also increased in 6-week ozone-exposed mice (Pinart et al. 2013). Upregulation of inflammatory cytokines and chemokines secondary to O_3_ exposure was also observed by Borthwick (2016).

In the present study, the increased gene expression of inflammatory cytokines was associated with inflammatory cell infiltration as detected in histopathological results. Our results were in agreement with that of Michaudel et al. (2016) and were explained by Mathews et al. (2014) and Fei et al. (2017) as chronic lung exposure to O_3_ caused pulmonary inflammation due to γδ T cells and TNFα-dependent recruitment of IL-17A. Fakhrzadeh et al. (2004) also added that O_3_ induces the production of nitric oxide, TNF-α leading to tissue injury, which is dependent on NF-kB p50. According to Bhalla et al. (2002), neutralizing TNF-α antibodies were found to reduce neutrophil recruitment in BALF together with decreased expression levels of IL-1α, IL-6, and IL-10 in animals exposed to O_3_.

Our findings were also similar to that of Nery-Flores et al. (2018) who proved that both acute and chronic exposure to O_3_ caused lipid peroxidation and protein oxidation in the neuronal tissue, together with increased serum levels of IL-1β, IL-6, and TNF-α in the hippocampus of rats. Xu et al. (2019) and Wiegman et al. (2020) elucidated that oxidative stress is the key mechanism underlying O_3_-induced lung injury. Chronic O_3_ exposure activates oxidative pathways resulting in chronic bronchial and bronchiolar inflammation and cell death as presented in our results by necrotic bronchiolar epithelium with some desquamated cells in the lumen of bronchioles. This can be explained by reaction of O_3_ with components of the fluid lining the air ways leading to the generation of reactive oxygen species (ROS) which induce oxidative stress, inflammation, and bronchiolar epithelial injury (Bromberg 2016). O_3_ induces lung injury and inflammation via the production of pro-inflammatory oxysterols (Speen et al. 2016) and suppression of pro-resolving lipid mediators (Kilburg-Basnyat et al. 2018).

In the present study, not only the airways were injured but the alveolar tissue was also severely injured in the form of collapsed alveoli with thickened interalveolar septae; the interstitium contained inflammatory cellular infiltration, congested blood vessels, and extravasated blood. According to Pulfer et al. (2005) and Kosmidir et al. (2010), alveolar injury occurred secondary to lipid peroxidation as O_3_ reacts with the phospholipids and cholesterol present in the cell membranes of alveolar epithelial cells and with lung surfactant, generating cytotoxic products with inflammatory cell recruitment. Tan et al. (2019) explained O_3_-induced cell injury by disruption of the integrity of the airway epithelial barrier through the affection of tight junction proteins. Kim et al. (2018) observed that the significant breaks in the tight junctions around the cells were associated with increased ROS, IL-1, 4, and 18 and TNF-α. So, tight junction affection is in part mediated by ROS-dependent mechanism. This disintegration causes a drop in the trans-electrical resistance between adjacent cells leading to cell death. Furthermore, it allows irritants, pathogens, and allergens to further aggravate epithelial injury (Wang et al. 2019). Affection of the tight junctions may explain in part the congestion observed in the interalveolar septa and interstitium of our study as a result of the induced inflammatory response according to Piontek et al. (2011).

In the present study, we detected a significant increase in the mean area percent of collagen fibers in the O_3_-treated rats as compared to the control and hypoxic MSCs groups. Collagen deposition was evident both in the interstitial tissue and around the bronchioles and blood vessels as evidenced by histochemical examination. Our findings were in accordance with that of Michaudel et al. (2016). Chronic inflammation resulting from continuous O_3_ exposure leads to fibroblast activation and proliferation associated with increased extracellular matrix deposition (Fehrenbach et al. 2017). Minor and Proud (2017) mentioned that bronchial epithelium secretes many inflammatory mediators allowing the interaction of these epithelial cells with interstitial fibroblasts mediating a process of epithelial to mesenchymal or fibroblast-myofibroblast transition, causing remodeling of the airways. Wang et al. (2019) demonstrated that injured bronchial epithelial cells release TGF-β which enhances the activity of nearby fibroblasts leading to an increase in collagen deposition.

iNOS is one of the key enzymes involved in the generation of cytotoxic and proinflammatory reactive nitrogen species implicated in the development of lung injury secondary to inhaled irritants (Laskin et al. 2011). In the present study, we proved a significant increase in iNOS immune expression in O_3_-treated group comparable to control and MSC-treated groups. During inflammation, iNOS expression is observed in several cell types, including macrophages, neutrophils, eosinophils, as well as in epithelial cells lining the airways (King et al. 2018). iNOS induces nitric oxide (NO) that contributes to both airway and distal lung parenchyma injury, inflammatory process, and extracellular matrix remodeling (Pigati et al. 2015).

From the previously mentioned mechanisms underlying O_3_-induced lung injury, we can infer that increased inflammatory cytokines, together with decreased antioxidant Nrf2 and enhanced iNOS expression, all contributed to cellular insult and apoptosis which was confirmed in our study by significant increased expression of anti-caspase 3 antibodies in lung tissue of O_3_-exposed rats. In line with our results, Rodríguez-Martínez et al. (2016) reported a significant increase in caspase 12 immune expression after 90 days of O_3_ exposure together with nuclear chromatin condensation in the rat hippocampus. They attributed it to chronic oxidative stress on the endoplasmic reticulum (ER) which triggers cell apoptosis via caspase activation. Caspase 12 (present in the outer membrane of ER) ultimately activates caspase 3, leading to apoptotic cell death (Hitomi et al. 2004). Holze et al. (2018) also reported apoptosis-like cell death induced by ROS (oxeiptosis) in O_3_-exposed mice lungs. Increased caspase-3 and apoptosis protease activating factor-1 immunoreactivity were also detected in the airways, alveolar epithelium, and macrophages of 3- and 6-week O_3_-exposed mice with inflammatory cell infiltration and increased collagen deposition secondary to excessive ROS and NLRP3 inflammasome production (Xu et al. 2019).

TEM examination further clarified the adverse effects of chronic O_3_ exposure on the blood air barrier, type II pneumocytes, and alveolar macrophages. According to Weigman et al. (2015), O_3_ exposure leads to mitochondrial damage induced by oxidative stress and accompanied by a reduced mitochondrial membrane potential. Rodriguez-Martinez et al. (2016) also reported mitochondrial dysfunction secondary to reactive oxygen and nitrogen species production. Dysfunctional mitochondria alter gene expression, immune response, cell metabolism, proliferation, and apoptosis (Praksh et al. 2017). Macrophages from O_3_-exposed lungs also undergo cell death by induction of apoptosis markers (cleaved caspase-9) and autophagy (beclin-1) according to Sunil et al. (2015).

In our study, we assessed the ameliorative effect of MSCs on lung injury induced by O_3_. MSCs are superior to embryonic stem cells or induced pluripotent stem cells due to their improved safety profile and non-existent ethical concerns. In addition, they are immune-privileged and do not trigger the host response as they are less sensitive to pro-inflammatory IFN-γ-induced HLA-II expression (Yang and Jia 2014; Gao et al. 2016). MSCs perform their function by secreting different growth factors responsible for tissue repair and regeneration such as angiopoietin 1, hepatocyte growth factor, keratinocyte growth factor, vascular endothelial growth factor alpha, and epidermal growth factor (Bernard et al. 2018).

In the present work, MSCs were administered to rats by IV injection in the tail vein and ameliorated both biochemical and histomorphological changes of the lung tissue. In previous research studies, we used systemically injected MSCs by IV route and proved their ameliorative effects on induced pathological changes in different rat and mice tissues including the cerebellum (Ahmed et al. 2017), the retina (Mohamed et al. 2017), the sciatic nerve (Abdelrahman et al. 2018), and the liver (Abdel Aal et al. 2019). It can be explained by MSC migration and homing that are promoted by chemo-attractants secreted by injured tissues and vascular endothelial cells (Castanheira et al. 2008).

MSCs’ therapeutic actions depend on paracrine production of neurotrophic and angiogenic factors besides immunomodulatory and anti-inflammatory activity, respectively (Waterman et al. 2012). According to Cooney et al. (2016), MSCs alter the inflammatory response by shifting the cytokine profile to an anti-inflammatory phenotype. Another explanation of the beneficial effects of IV injected MSCs on the host immune response is by increasing the release of prostaglandin E2 from the BM-derived MSCs acting on the EP2 and EP4 receptors of the macrophages and by stimulating the production and release of IL10 (Németh et al. 2009). According to Mahrouf-Yorgov et al. (2017), MSCs act by several mechanisms such as modulating the inflammatory response, enhancing antioxidant defenses, and augmenting cellular respiration and mitochondrial functions. They even can donate their mitochondria to protect damaged cells. They also decrease expression of ROS-producing enzymes, iNOS, and neutrophil infiltration.

MSC-treated groups in the present study revealed improvement in biochemical parameters with greater improvement being observed in the hypoxia-preconditioned MSCs. Regarding histopathological examination, normoxia MSC-treated group showed amelioration in the lung morphology apart from some darkly stained bronchiolar cells nuclei with thickened interalveolar septae and inflammatory cellular infiltration in some sections. According to Chen et al. (2006), MSCs are highly resistant to oxidative insult and can scavenge free radicals. They are resistant to oxidative and nitrosative stimuli as evidenced by significant upregulation in Nrf2 expression and significant downregulation of iNOS in our study that could be attributed to the antioxidant effects of MSCs and thus explains amelioration in lung morphology together with decreased expression of caspase 3.

Our results were also in accordance with Bernardo and Fibbe (2013) and Saldana et al. (2019). They stated that MSCs enhance tissue repair and regeneration by immune response modulation, rather than by replacing damaged cells. According to Galli et al. (2011), macrophages exhibit functional repolarization and shifting from the pro-inflammatory (M1) phenotype to an anti-inflammatory (M2) phenotype during tissue repair as M2 type secretes less cytokines and enhances tissue repair. In addition, Ye and colleagues proved that BMSCs can affect endogenous lung stem cells (club cells) via cytokines as well as vesicles and activate the Notch signaling thus enhancing the proliferation of club cells in phosgene induced lung injury (Ye et al. 2019).

Pre-treatment of MSCs boosts their survival, paracrine, and immunomodulatory traits according to Abdelrahman et al. (2018). Preconditioning of MSCs involves the ex vivo treatment with both chemical and physical factors via specifically designed environment to enhance the intrinsic therapeutic properties of MSCs (Ocansey et al. 2020).

We have proved that hypoxia pretreatment enhanced MSCs homing in lung tissue as evidenced by fluorescent imaging of PKH26-labeled stem cells together with immunolocalization of CD105-positive MSCs in lung tissue. In addition, MTT assay demonstrated that hypoxia pretreatment improved MSC survival than normoxic MSCs. Our results were in accordance with that of Lan et al. (2015) who proved that hypoxic preconditioning increases survival time of engrafted MSCs.

Taken together, increased stem cells homing and survival can explain the amelioration of lung tissue in hypoxic MSC group of our study regarding the histological structure, the mean area percent of collagen, caspase 3, and iNOS immune expression that were comparable to the control group. According to Sara and Weiss (2020), MSC-based therapies for severe lung diseases have demonstrated promising results in experimental lung models; however, the desired outcomes of MSC-based therapy can be further improved via MSC modifications (Ocansey et al. 2020). Hypoxia-treated MSCs express more anti-apoptotic proteins, IL-8, and IL-6 according to Chen et al. (2014), as well as IL-10 and FasL (Jiang et al. 2015). Hypoxic preconditioning showed greater MSC cellular complexity and decreased tendency to autophagy, therefore enhancing their survival according to Pezzi et al. (2017).

Bernard et al. (2018) reported that, under hypoxic conditions, keratinocyte and hepatocyte growth factors that are released from MSCs can protect alveolar epithelial cells from apoptosis by stabilization of endogenous Bcl-2 and inhibition of both of HIF1α protein expression and ROS production. Hypoxia-preconditioning of MSCs strengthens their paracrine abilities and enhances their migratory capacity (Lee et al. 2017; Wang et al. 2017). Hypoxic environment enhances the proliferation of MSCs, inhibits apoptosis, and facilitates migration and chemotaxis (Wang et al. 2021). Hypoxia induces the expression of cytoprotective genes and also encourages the secretion of anti-inflammatory, anti-apoptotic, and anti-fibrotic factors. Intratracheal instillation of hypoxia-pretreated MSCs significantly ameliorated lung fibrosis by attenuating the extracellular matrix production. Downregulation of inflammatory factors and improving pulmonary respiratory functions were also evident (Lan et al. 2015).

## Conclusion and recommendations

From the previous results, we can conclude that chronic O_3_ exposure in polluted urban areas has deleterious health effects on the airways and the entire lung tissue via enhancing inflammatory reaction and oxidative and nitrosative stress. MSCs represent promising intervention to overcome chronic lung injury by immune-modulatory, antioxidant, and anti-apoptotic effects. Hypoxia pretreatment of MSCs enhances their survival, homing, and therapeutic potential on O_3_-inducd chronic lung injury. More studies are recommended to delineate other mechanisms of action of hypoxia-pretreated MSCs before being applied on patients with chronic lung diseases.
